# Research on SR/Frit Composites: A Novel Low-Temperature Ceramifiable Expandable Flame-Retardant Material

**DOI:** 10.3390/ma15092961

**Published:** 2022-04-19

**Authors:** Hongwei Zhu, Jianhua Li

**Affiliations:** School of Police Equipment and Technology, Chinese People’s Police University, Langfang 065000, China; zhuhongwei@cppu.edu.cn

**Keywords:** frits, silicone rubber (SR), ceramization, expandable and ceramifying performance, ceramization temperature

## Abstract

Silicone rubber (SR) exhibits unique flame-retardant and fireproof properties and can be ceramized at high temperatures to cover the surface of an object that needs fire protection. In this paper, the influence of low-melting-point frit content on the expandable performance of SR has been investigated, and a decrease in the ceramization temperature with an increase in the frit content has been observed. The sample began to expand at 850 °C, and an expansion of 157% and compressive strength of 1.99 MPa were attained at 950 °C. The increased frit content resulted in a larger liquid phase, which covered the surface of the matrix owing to surface tension. This made the escape of small-molecule gaseous substances generated by decomposition difficult: this resulted in the expansion of the SR matrix. The relationships between composite compositions and expansibility as well as the degree of ceramization were also explored through performance tests. It was found that the formation of eutectic substances between frits and the matrix resulted in a decrease in the temperature of ceramization, which in turn contributed to the formation of highly intumescent ceramifiable SR.

## 1. Introduction

**Figure 1:**
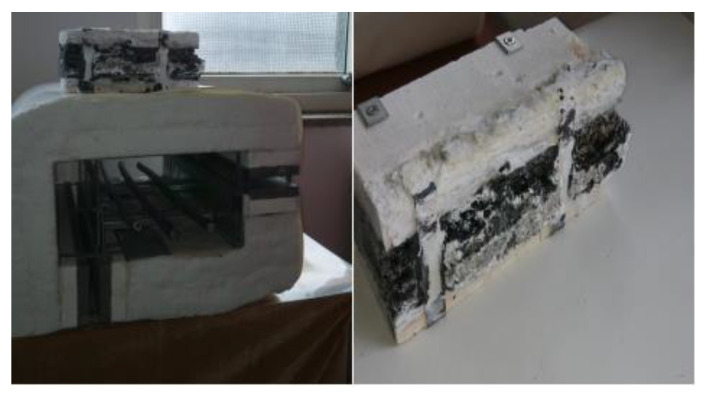
Ceramifiable IFR protector and the IFR protection effect.

The concept of polymer–ceramic composites was proposed by Prof. Hanu et al. [1] Subsequently, lots of work was conducted to obtain CSR and different fillers, such as porcelain fillers, melting aids, reinforcing agents, and flame-retardant agents, were added to improve the performance [1,2,3,4,5,6]. Pędzich et al. [7] studied the effect of wollastonite, bentonite, and kaolin on CSR when three minerals were used as fillers. With the development of silicone rubber (SR), the market has been provided with an exemplary product that actually meets the application needs that cannot be met by other elastomers. The main advantages of SR are its ability to function in harsh environments, resistance to high and low temperatures, electrical insulation properties that are not susceptible to environmental influences, and inertness to human biological systems. Consequently, SR is used in a wide range of applications across industries, including medical and healthcare, aerospace, electronics and electrical, and wire and cable [8,9,10]. When there is a risk of fire in key infrastructural installations, protection of various cables with a safe expandable sealing system is particularly important. Power cables are critical units in any installation that require special fire protection in a single fire zone. The expandable flame retardant (IFR) module comprises top and bottom modules that are normally open to facilitate heat dissipation, whereas they expand in the event of a fire and seal the opening to protect the cable. A cross-sectional view of the power cable protector is shown in Figure 1 (image on the right), which shows the IFR protection effect.

The use of ceramifiable silicone rubber (CSR) in applications such as fire protection of wires and cables requires the product to be expandable in order to seal the outlet while maintaining its integrity at a high temperature (Figure 1).

**Figure 2:**
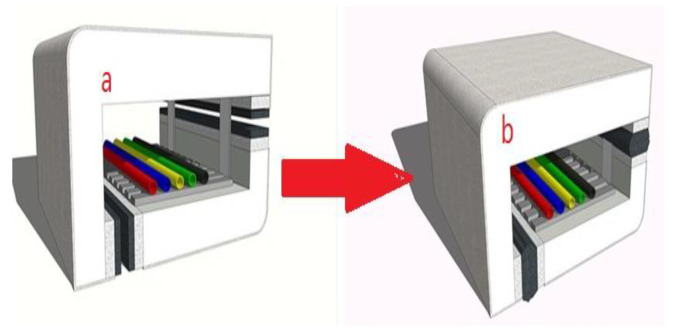
Sectional views of the cable tray protector before and after thermal expansion of the CSR–IFR module: (**a**) daily state and (**b**) closed state in the event of a fire.

The expansion and ceramization mechanisms of CSR for retarding flame are illustrated in Figure 2. An active flame-retardant effect can be achieved through effective control of the expansion temperature and the extent of expansion.

Therefore, the fire-resistant CSR is a “revolutionary” product that can serve as a breakthrough material for fireproofing and fire protection. Furthermore, it offers a completely new method for manufacturing fireproof and heat insulation materials as well as related products. Particularly, CSR with expandable properties offers superior performance in terms of fire protection. Researchers have conducted studies on IFR materials, which are favored for their low smoke emission, zero halogen content, low toxicity, and other such characteristics. Some of the most commonly used IFR systems include ammonium polyphosphate (APP), melamine (MEL), pentaerythritol (PER), and expandable graphite (EG) [9,10,11,12,13]. IFR systems provide good heat insulation and flame-retardant properties, and studies on expandable CSR systems have been reported [14,15]. 

However, the high ceramization and expansion temperatures of CSR materials restricts their application in many areas. In the case of the power cable tray protector application presented above, low expansion and ceramization temperatures are required so that the protective action is initiated early to separate the flame before the fire spreads. Therefore, the study of reducing the expansion and ceramization temperatures is of great significance for developing high-performance ablation resistance and thermal protection systems.

Frits, a type of amorphous powders, are the most commonly used flux for CSR. They are primarily composed of SiO_2_, Al_2_O_3_, sodium oxide (NaO), phosphorus pentoxide (P_2_O_5_), and other metal oxides. In the early days, the melting point of low-melting-point frits was mainly adjusted by changing the content of P_2_O_5_, which is a toxic substance. Therefore, they were gradually replaced by lead-free low-melting-point frits, such as the Bi_2_O_3_–ZnO–B_2_O_3_ and Bi_2_O_3_–B_2_O_3_–BaO systems, later on [16,17,18,19]. Hanu et al. [16] blended mica, frits, FeO, and other fillers with SR to prepare CSR with high-temperature resistance for wire and cable applications and analyzed its thermal stability and heat release rate. Their experimental results demonstrate that mica and FeO can reduce the heat release rate of the material and enhance the thermal stability of the matrix. In addition, because the addition of frits decreases the thermal stability of SR, their content in the mix needs to be limited within 5–20 wt%, even though frits can promote the ceramization process. In a study by Zeng et al. [17], different ratios of frit contents were added to the SR matrix to obtain CSR and the matrix was analyzed for its mechanical and ceramization properties. Their experiment demonstrated that a dense and hard ceramic material could be obtained by calcination at 700 °C for a certain period in a muffle furnace, and it was also found that increasing the frit content would worsen the mechanical properties and fluidity. Shao et al. [18] used low-melting-point frits as fillers that enabled ceramization and blended them with SR to prepare CSR. Moreover, they investigated the influence of frit content on the mechanical and ceramization properties of SR and learned that although the frit content had no significant influence on SR’s tensile strength, an increase in the frit content effectively lowered the ceramization temperature, whereas an increase in ceramization temperature enhanced the ceramization strength. Further, through scanning electron microscopy (SEM), it was observed that the internal compactness of the ceramic product deteriorated on increasing the frit content.

Flame-retardant polymer materials are an update and the future for the high-end-manufacturing field, for example, 3D printing developments [20]. By studying the influence of the frit content on the expandable and ceramification performances of SR, this paper explored the preparation of composite materials that can expand and ceramize simultaneously to meet the flame-retardant performance requirements of special materials used under high-temperature conditions. Expandable SR materials were prepared, and the relationships between their composition and expandability as well as the degree of ceramization were investigated. The findings of this research are of great theoretical importance for the preparation of expandable SR through the ceramization process and can promote the research and development of novel low-temperature ceramifiable IFR materials.

## 2. Experiments

### 2.1. Experimental Raw Materials

The raw materials used were methyl vinyl silicone rubber raw rubber (SR): DJ-2250; white carbon black by precipitation method; mica: average particle size of 800 mesh; aluminum hypophosphite (AHP); calcium carbonate; and frits: DZ655, with a melting point of 400 °C and fineness of 3000 mesh.

Required fillers are weighed according to the formulation presented in Table 1. AHP is dried in a drying oven at 80 °C for 12 h, and the required fillers are weighed in accordance with the formulation in Table 1, blended evenly, and set aside for later use. The blending procedure is performed in an open mill at room temperature. First, raw SR is mixed using a two-roll mill. Then, fillers are uniformly blended. When the blending is uniform, approximately 2.5 g of double 2,4 vulcanizing agent (DCBP) is added and the blending is continued for 3 to 5 min. After increasing the distance between the two rollers, the thin sheet is passed through. The process outlined above takes approximately 15 to 20 min.

After the sample has been uniformly mixed, it is vulcanized for 12 to 15 min on a flat vulcanizer at a temperature of 160 °C and pressure of 15 MPa in the specified mold. During secondary vulcanization, the sample is placed in a 200 °C blast constant temperature drying oven for 5 h or more. The sample is then cooled and prepared into the desired sample size.

### 2.2. Experimental Instruments and Apparatus

The main instruments and apparatus used in the experiments are listed in Table 2.

### 2.3. Performance Tests

The GB/T 10707-2008 standard is adopted for testing the limiting oxygen index (LOI) for the SR composite and a sample size of 80 × 6.5 × 3 mm^3^ is used. The flow rate of the oxygen–nitrogen mixed gas is adjusted to 10 L/min. The oxygen content in the gas flow is adjusted to burn the sample strip, and the LOI data are recorded according to the national standard. The oxygen concentration displayed on the instrument indicates the current LOI. During the sample strip test, the burning condition of the sample strip is also recorded.

The vertical burning test also follows the standard GB/T 10707-2008, and a sample size of 130 × 13 × 3 mm^3^ is used. The sample is clamped vertically on the fixture, a piece of absorbent cotton is placed directly under the sample, and a flame is applied for a specified period to burn the sample. After that, the fire source is removed and the flame burning and flameless burning times are recorded. The presence of drips and whether the drips can ignite absorbent cotton are also noted to determine the combustion level of the sample. The following are the specific judgment criteria.

Thermogravimetric analysis can be used to evaluate the thermal stability of SR composite samples. With a thermal gravimetric analyzer (Shimadzu, Japan), samples with a mass of 5–8 mg are tested in an atmosphere of N_2_. During the test, the temperature rises from room temperature to 800 °C at a rate of 10 °C/min to generate the thermogravimetry (TG) curves.

Mechanical performance tests of SR composites include the tests of tensile strength, elongation at break, and Shore hardness. Tensile strength and elongation at break are measured according to GB/T 528-2009. These tests are conducted on a universal tensile machine. The sample is dumbbell shaped, measuring 115 mm in length. The clamps are spaced 80 mm apart; the narrow part is 33 mm long and 6 mm wide; the gauge length is 25 mm; the thickness is 3 mm; the tensile rate is 500 mm/min. Shore hardness is measured in accordance with GB/T 531.1-2008.

The SEM (Hitachi, Japan) is primarily used to observe the microscopic morphology of the CSR specimens and the microscopic expansions of expandable CSR specimens at different temperatures. The samples must be sputtered with gold in the test, and an acceleration voltage of 20 kV is employed.

Tests of ceramic strength primarily involve the measurement of compressive strength of the ceramic bodies calcined at different temperatures within a muffle furnace. The dimensions of the SR cylindrical samples are 20 mm diameter and 20 mm length. The samples are calcined for 30 min in a muffle furnace at predetermined temperatures and then cooled to room temperature. Subsequently, compression tests are performed on the samples. As per GB/T 8489-2006, the strength of the ceramic is characterized by the force required to destroy the sample. The compressive strength is calculated by the following equation:

(1) $$\sigma_{P}=\frac{P}{A}$$

where $\sigma_{P}$ is the compressive strength of the material in MPa; P is the critical load, i.e., the maximum pressure the material can withstand in Newtons; and *A* is the cross-sectional area of the sample in mm^2^: $A=\pi d^{2}/4$. When calculating the compressive strength, the result is reserved to integer digits; if the value is less than 100 MPa, three significant figures are reserved.

To evaluate the volume variation in the IFR–SR composite, SR composites are prepared in 20 × 20 × 3 mm^3^ cuboids and calcined in a muffle furnace. For the expansion tests of the CSR, cylindrical samples of the SR composites with a size of 20 mm diameter and 20 mm length are prepared and then calcined at predetermined temperatures in a muffle furnace for 30 min. To calculate the volume, the diameter and length are measured before and after calcination using a vernier caliper. The volume variation is then determined according to the following equation:

(2) $$\mathrm{Volume}\;\mathrm{variation}\;(\%)=\frac{\left(\mathrm{volume}\right)\mathrm{residue}-\left(\mathrm{volume}\right)\mathrm{sample}}{\left(\mathrm{volume}\right)\mathrm{sample}}\;\times \;100\%$$

## 3. Results and Discussion

### 3.1. LOI and UL-94 Tests of the SR/Frit Composites

Details of the changes in the flame-retardant properties of SR composites corresponding to the frit content are presented in Table 3. It can be observed that the sample SR’s LOI was 30.9% with no flux added, which did not change significantly with flux addition. This is because the flux is composed of low-melting-point frits and primarily used to form silicon and boron oxides and other substances via melting, polymerizing, and crystallizing at high temperatures after the fillers have been evenly mixed. The frits also help reduce the melting point of the filler by forming eutectic points with other inorganic fillers at high temperatures, although the filler has a limited effect on the flame-retardant properties of SR. However, a higher content causes a marginal rise in LOI primarily due to an increase in inorganic fillers. It can be seen from Table 3 that the UL-94 value keeps class 0, showing that the SR composite has a good ability to extinguish after ignition, which is because the addition of inorganic substances enhances the flame-retardant and heat insulation ability.

### 3.2. Thermogravimetric Analysis of SR/Frits Composites

**Figure 3:**
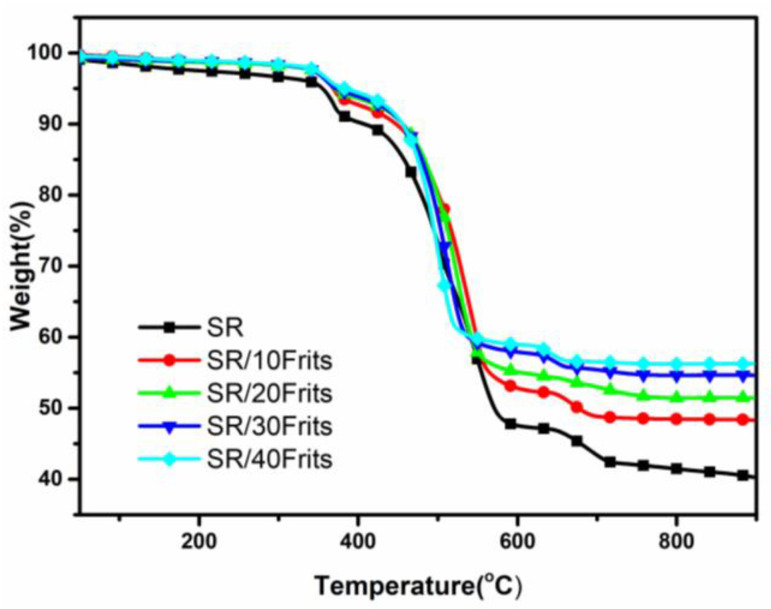
TG curves of SR/frit composites.

The influence of the frit content on the thermal stability of SR composites is investigated using thermogravimetric analysis. The TG curves for different frit contents are presented in Figure 3. The test temperature ranges from room temperature to 900 °C. According to Figure 3, the decomposition of SR composites can be divided into three stages, of which the first two stages primarily resulted from the presence of AHP. As shown in Figure 3, the 5%-mass decomposition temperature reduced upon the addition of frits; for example, the addition of 40 parts per hundred (phr) of frits lowered the 5%-mass decomposition temperature from 394 to 368 °C, the reason for this being that certain metal oxides in frits catalyze the cross-linking of SR, making it degrade at relatively lower temperatures [21]. The results suggest that the addition of frits reduces the thermal stability of SR composites and the maximum decomposition temperature of SR also decreases with an increase in the frit content. This indicates that the degradation of the molecular backbone is also affected. The experimental results confirm that the residue content in SR increased gradually from 40.27 to 56.25%, corresponding to an increase in the frit content. This can be attributed to the fact that frits exhibit a certain level of chemical stability and an increase in the frit content decreases the proportion of easily decomposable organic matter in SR [21].

### 3.3. Influence of Low-Melting-Point Frit Content on the Macroscopic Morphology of Ablation Residues

**Figure 4:**
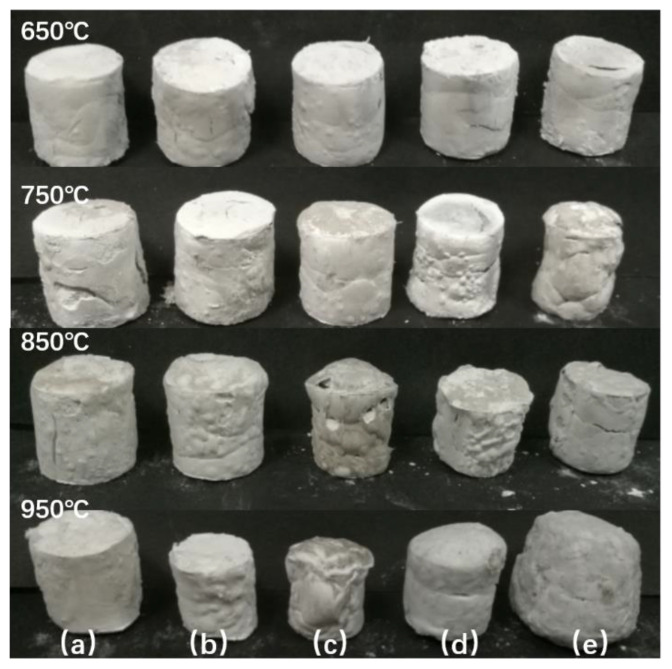
Macroscopic morphology of the residues at different temperatures of (**a**) SR, (**b**) SR/10Frits, (**c**) SR/20Frits, (**d**) SR/30Frits, and (**e**) SR/40Frits.

The influence that a variation in the frit content has on the macroscopic morphology of expandable CSR composites is presented in Figure 4. The figure shows that at 650 °C, the surfaces of different SR samples were essentially the same and the surface was a gray-white smooth cylinder, whereas at 750 °C, the ablated SR cylinder began to shrink and became smaller owing to the presence of frits: the cylinder surface was deformed and no longer smooth. Because the increase in the frit content with a low melting point caused the liquid phase to increase during ablation, shrinkage of the cylinder occurred and the cylinder surfaces gradually became irregular between 850 and 950 °C. However, after the ablation process, the volume of the small cylinder contracted first and then expanded because at high temperatures, the liquid phase generated by the melting of frits causes the volume of the small cylinder to contract. Due to the increased content of frits and the high-temperature environment, the liquid phase not only formed a bridge between the powders but also enclosed the surface of the sample. This made it difficult for the gases generated by the decomposition of the inorganic fillers at high temperatures to escape from the matrix. Eventually, this led to a volume expansion of ablation residues. On dissection of the expanded sample, the formation of a dense ceramic structure that could self-support or even resist external erosion forces could be observed on the outside, whereas the formation of a fluffy, porous, hard carbon layer was found on the inside [14,15].

**Figure 5:**
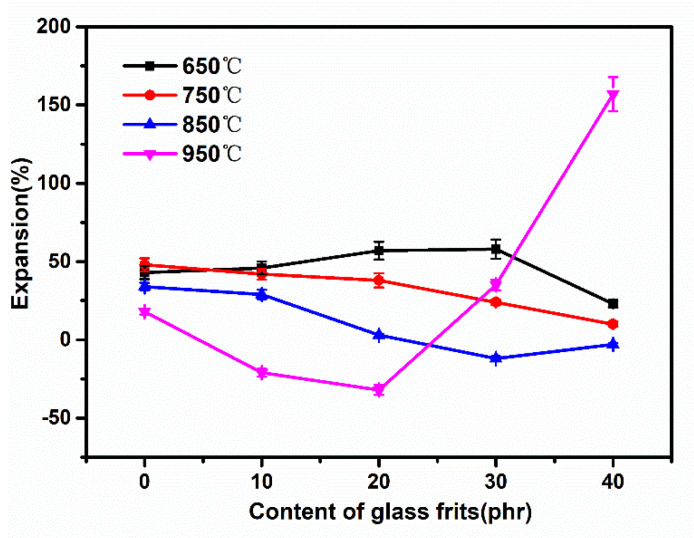
Volume variations in the residues at different temperatures of silicone composites.

Figure 5 shows a line graph plotted based on the volume variations. No obvious variation pattern was observed at 650 °C because the liquid phase formed by the melting of frits was relatively limited. The volume exhibited a downward trend when the frit content increased to 20 phr because as the frit content increased, correspondingly, the proportion of the molten liquid phase also increased, which led to a decrease in volume. The volume decreased from the initial 48 to 10% with an increase in the frit content at 750 °C. Nevertheless, the volume remained in an expanded state when compared to the original sample because the decomposition of the SR matrix releases small-molecule organics and other gases and the decomposition of CaCO_3_ at this temperature releases gases, such as carbon dioxide, and the combined effect of both leads to the expansion of the SR matrix. At 850 °C, it could be observed that initially, the volume contracted as the frit content increased, whereas when the frit content increased to 40 phr, the trend reversed and expansion could be observed. The main reason for this phenomenon is that the increase in the liquid phase makes it difficult for the gases to escape. On further analysis of the variation in volume at 950 °C, more noticeable changes were observed. Initially, the volume contracted with the addition of frits. Subsequently, the addition of 20 phr of frits contracted the volume by 32%; the addition of 30 phr of frits expanded the volume to 35%; and the addition of 40 phr of frits expanded the volume further, to 157%. Taking into consideration the influence of factors such as temperature and the frit content, as well as the fact that there was no weight loss between 800 and 900 °C in the thermogravimetric analysis, it is speculated that the main cause for the volumetric expansion was an increase in the liquid-phase composition in the matrix consequent to the increase in both frit content and temperature. The formation of a massive liquid phase prevented the escape of the gases generated during the decomposition of the matrix and other fillers and trapped them within the matrix. Hence, it can be concluded that an increase in both temperature and frit content can lead to an increase in the liquid phase. Accordingly, at 850 °C, the volume began to expand after 40 phr of frits was added; at 950 °C, the interaction between the two factors further increased the liquid phase: essentially, the volume began to expand after 30 phr of frits was added.

### 3.4. Influence of Low-Melting-Point Frit Content on the Microscopic Volume of Ablation Residues

**Figure 6:**
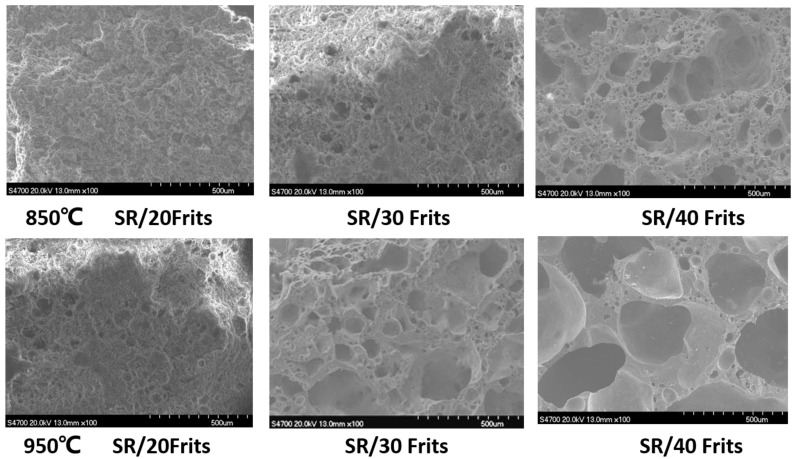
Volume variations in the residues of micromorphology at different temperature of silicone composites.

The irregular volume variation patterns observed at 850 and 950 °C, discussed in Section 3.3, were further examined at a microscopic level, as shown in Figure 6: samples SR/20Frits, SR/30Frits, and SR/40Frits were selected for SEM examination. As depicted in the images, unevenly distributed pores could be observed in the matrix of the burned sample SR/20Frits at 850 °C at a magnification of 100×. In sample SR/30Frits, it could be observed that the pores became smaller, the pore distribution became more uniform, and the overall matrix appeared dense. This is because the higher frit content generated more liquid phases, which bonded the fillers together, along the gaps and cracks, thereby enhancing the sintering effect. With a further increase in the frit content, the presence of liquid bridges became more apparent in sample SR/40Frits. Furthermore, larger pores and an uneven pore distribution were observed in the sample: the pores were more apparent at 950 °C than at 850 °C. An obvious liquid-phase structure could be observed in sample SR/20Frits, and the pores remained evenly distributed in the matrix; this distribution was most likely caused by liquid-phase connections. The pores present in sample SR/30Frits were evenly distributed on the surface of the ceramic-like substance connected by the liquid bridge, and there were also some relatively larger pores in the surrounding area. Sample SR/40Frits exhibited larger pores, as well as small, dense pores in the liquid bridge that most likely resulted from the expansion of small-molecule substances generated during the decomposition process of SR, which formed pores in the material. Because of the short duration of high temperatures, the densification effect was weak, which resulted in their retention in the form of pores in the matrix [22]. It was found that the expansion of sample SR/40Frits at 950 °C was caused primarily by too much liquid-phase composition, which caused pores in the matrix during the formation of liquid bridges.

Additionally, glassy borides and similar substances have a low viscosity at high temperatures and a large expansion coefficient, which causes the liquid-phase structures to accumulate readily on surfaces under the action of surface tension and heat flow [14,15].

### 3.5. Influence of Low-Melting-Point Frit Content on the Compressive Strength of Ablation Residues

**Figure 7:**
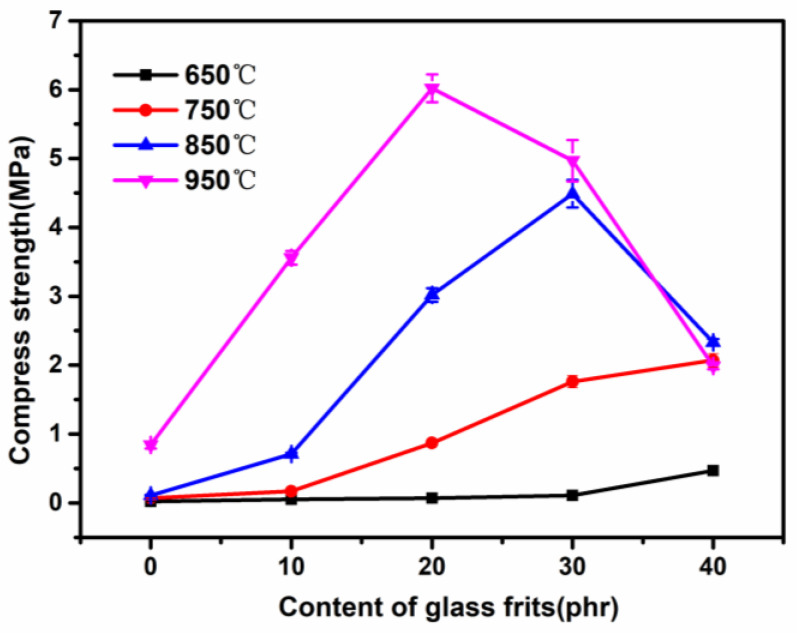
Compression strength of the residues of the silicone composites at different temperatures.

Figure 7 shows the influence of the frit content on the compressive strength of the ablation residues of SR composites. The figure shows that at 850 °C and below, the compressive strength increased with the increase in the frit content. This could be attributed to the fact that a higher frit content caused the liquid phase to increase at higher temperatures and the sintering effect to become stronger, which led to a more rapid sintering process. A higher sintering degree thus resulted in a higher strength of the ceramic product [23]. At 950 °C, the compressive strength dropped to 2.97 MPa after the addition of 30 phr of frits. This is due to the volume expansion of pores caused by the decomposition of low-melting-point oxides. Despite the expansion, the compressive strength still reached 1.99 MPa when 40 phr of frits was added, which is much higher than that of the sample SR without frits. This also indicates that flux is crucial for the preparation of CSR.

### 3.6. Influence of Low-Melting-Point Frit Content on the Microscopic Morphology of Ablation Residues

**Figure 8:**
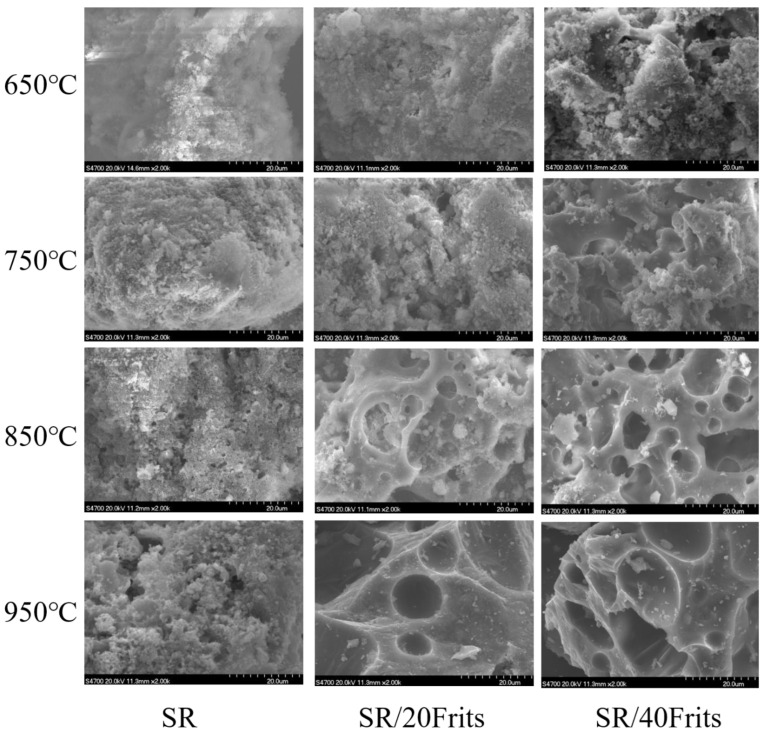
SEM images of the ceramics of SR, SR/20Frits, and SR/40Frits at different temperatures.

Figure 8 shows the microstructures of samples SR, SR/20Frits, and SR/40Frits after ablation at different temperatures, and it is evident from the images that the same sample exhibited different microstructures at different temperatures. The fillers in sample SR were in a powder state at 650 °C, and the surface was relatively flat. As the temperature continued to rise, a small amount of liquid phase was observed at 850 °C. This is the liquid phase resulting from the partial melting and the eutectic reaction of the ceramification fillers. When the temperature increased further, the liquid phase also increased. At 650 °C, sample SR/20Frits appeared to be stronger than sample SR. The melting of the frits caused the formation of a small amount of crystal phase on the surface. On reaching 850 °C, the ceramic skeleton resulting from the liquid bridge structure formed by the liquid phase as well as the presence of pores can be clearly observed. With an increasing temperature, the liquid phase flowed more completely, resulting in fewer and larger pores and the formation of bulk ceramic eventually. The SR/40Frits sample exhibited an obvious liquid-phase structure at 650 °C, and a liquid bridge structure had already developed at 750 °C. The liquid phase formed during the melting of frits penetrated the matrix and bonded the fillers together. A large number of pores could be observed in the ablated sample at 850 °C, which became more apparent at 950 °C. It can be seen that this phenomenon corresponds well to the macroscopically observed volume variation mentioned previously. It is primarily caused by the large amount of liquid phase covering the surface, which made it difficult for the formed gases to escape. There were also liquid bridges that were mutually connected by the flow of the liquid phase, which eventually led to the formation of pores.

Furthermore, based on a careful analysis of Figure 8, it could be concluded that an increase in the frit content can effectively reduce the ceramization temperature. The low-temperature ceramization process is primarily determined by the sintering and bonding of the frits, and an increase in the frit content would enhance the sintering effect, which in turn would result in a lower ceramization temperature [24,25,26,27].

### 3.7. Mechanical Performance Tests of SR/Frit Composites

Table 4 shows the test results of the impact of frit addition on the mechanical properties of the expandable CSR. Without flux, the sample’s tensile strength was 4.7 MPa and its elongation at break was 302%. With the addition of frits, the mechanical strength greatly reduced; for example, the addition of 40 phr of flux reduced the tensile strength to 2.6 MPa and the elongation at break decreased from 302 to 176%. The mechanical properties of the SR matrix were impaired owing to the addition of frits, while simultaneously the hardness was also found to increase. Because frits are inorganic fillers, their hardness is greater than that of SR and so the overall hardness increases. In addition, frits are not compatible with the SR matrix and, hence, the overall mechanical properties were substantially compromised. As shown in Table 4, consequent to the addition of 40 phr of frits, the SR became hard, with the hardness increasing from 68 ± 1 to 75 ± 2. However, the tensile strength and the elongation at break both decreased, because of which, the SR exhibited low toughness. All this can be attributed to the addition of the frits.

### 3.8. Expansion and Ceramization Mechanism of SR/Frit Composites

**Figure 9:**
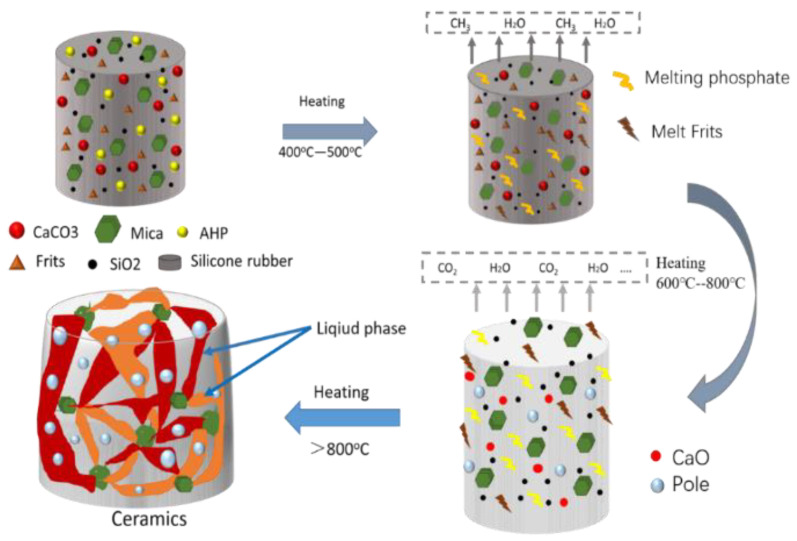
Ceramic expansion mechanism of SR/frits.

Figure 9 illustrates the expansion and ceramization mechanisms of the SR composite. At room temperature, the fillers are evenly dispersed in the SR matrix, and at lower temperatures (400–500 °C), the frits melt and primarily act as a bonding agent. However, because an increase in the frit content reduces the ceramization temperature, as discussed previously, a large amount of liquid phase will likely be produced at a lower temperature. At approximately 700 °C, CaCO_3_ begins to decompose and the SR matrix is essentially completely decomposed. The matrix fillers and the SiO_2_ formed by the decomposition of SR are connected by a liquid phase generated from the melting of the frits or the eutectic reaction [15]. Subsequently, when the temperature rises above 800 °C, the liquid phase increases, gathers on the surface of the matrix, and covers it, owing to the surface tension and heat flow. Some of the intermediate products generated by the reaction between the fillers, such as carbon dioxide and small-molecule gases, that are produced as a result of carbonate decomposition are unable to escape. Moreover, pores are created in the matrix by the flow, connection, and convergence of the liquid phase. Consequently, an expandable composite of CSR consisting of a dense outer layer and a fluffy, porous, and hard inner layer is formed.

## 4. Conclusions

The relationship between the frit content and properties such as the expansion and ceramization of SR was studied by preparing expandable CSR samples with different flux contents and analyzing them. Furthermore, the influence of the frit content on the composites’ properties, including volume variation at high temperatures, compressive strength, density, and mass loss, were investigated and characterized. It was observed that the sample with 40 phr of frits expanded 30 min after ablation in the muffle furnace at 850 °C and the expansion became more pronounced with an increase in temperature, subsequently reaching 157% at 950 °C. Test results of the density change and the compressive strength were in agreement with the volume variations. An expandable CSR composite with a dense outer layer and a fluffy, porous, and hard inner layer was successfully prepared by adding 40 phr of frits: it attained a compressive strength of 1.99 MPa at 950 °C. A flame-retardant performance test on the sample revealed that the frit content did not influence the flame-retardant properties of this formulation, whereas a significant negative influence on the mechanical properties was observed with the addition of 40 phr of frits. Further, it was observed that the addition of 6 g of methyl silicone oil to the matrix with SR/40Frits increased the mechanical strength by 42%, from 2.6 to 3.7 MPa, whereas the addition of a compatibilizer did not affect the expandable or ceramification properties of the composite. It is believable that intumescent flame-retardant ceramic silicone rubber materials will be deeply studied and widely used in various fields.

## Figures and Tables

**Table 1:** Formulations of CSR/frit composites.

Samples	SR	AHP	Mica	CaCO_3_	SiO_2_	Frits
SR	100	10	5	10	20	0
SR/10Frits	100	10	5	10	20	10
SR/20Frits	100	10	5	10	20	20
SR/30Frits	100	10	5	10	20	30
SR/40Frits	100	10	5	10	20	40

**Table 2:** Experimental instruments and apparatus.

Instrument/Apparatus	Model/Specification	Manufacturer
Vertical Burning Tester	JF-3	Nanjing Jiangning Analytical Instrument Co., Ltd., Nanjing, China
Oxygen Index Tester	JF-3	Nanjing Jiangning Analytical Instrument Co., Ltd., Nanjing, China
Universal Electronic Tensile Machine	WDW-50E	Jinan Shijin Group Company Ltd., Jinan, China
Thermal Gravimetric Analyzer	TAQ-500	Shimadzu, Kyoto, Japan
Scanning Electron Microscope	HITACHI-S4800	Hitachi, Tokyo, Japan
Shore Hardness Tester	Shore A	Shanghai Luchuan Measuring Tools Co., Ltd., Shanghai, China

**Table 3:** LOI and UL-94 results of SR/frit composites.

Samples	LOI	UL-94		
		T1	T2	
SR	30.9 ± 0.2	-	-	None
SR/10Frits	31.7 ± 0.1	0	6 ± 1	None
SR/20Frits	32.0 ± 0.3	0	0	None
SR/30Frits	32.3 ± 0.2	0	0	None
SR/40Frits	32.4 ± 0.1	0	0	None

**Table 4:** Tensile strength, elongation at break, and hardness of SR/frit composites.

Samples	Tensile Strength (MPa)	Elongation at Break (%)	Hardness
SR	4.7 ± 0.5	302 ± 30	68 ± 1
SR/10Frits	3.9 ± 0.4	257 ± 25	70 ± 2
SR/20Frits	3.6 ± 0.3	235 ± 25	72 ± 1
SR/30Frits	2.9 ± 0.3	218 ± 20	74 ± 1
SR/40Frits	2.6 ± 0.3	176 ± 20	75 ± 2

## Data Availability

The data used to support the findings of this study are included within the manuscript.
